# Screening and Evaluation of Deleterious SNPs in APOE Gene of Alzheimer's Disease

**DOI:** 10.1155/2012/480609

**Published:** 2012-03-13

**Authors:** Tariq Ahmad Masoodi, Sulaiman A. Al Shammari, May N. Al-Muammar, Adel A. Alhamdan

**Affiliations:** Health Care Development for Elderly Research Chair, Community Health Sciences Department, College of Applied Medical Sciences, King Saud University, P.O. Box 10219, Riyadh 11433, Saudi Arabia

## Abstract

*Introduction*. Apolipoprotein E (APOE) is an important risk factor for Alzheimer's disease (AD) and is present in 30–50% of patients who develop late-onset AD. Several single-nucleotide polymorphisms (SNPs) are present in APOE gene which act as the biomarkers for exploring the genetic basis of this disease. The objective of this study is to identify deleterious nsSNPs associated with APOE gene. *Methods*. The SNPs were retrieved from dbSNP. Using I-Mutant, protein stability change was calculated. The potentially functional nonsynonymous (ns) SNPs and their effect on protein was predicted by PolyPhen and SIFT, respectively. FASTSNP was used for functional analysis and estimation of risk score. The functional impact on the APOE protein was evaluated by using Swiss PDB viewer and NOMAD-Ref server. *Results*. Six nsSNPs were found to be least stable by I-Mutant 2.0 with DDG value of >−1.0. Four nsSNPs showed a highly deleterious tolerance index score of 0.00. Nine nsSNPs were found to be probably damaging with position-specific independent counts (PSICs) score of ≥2.0. Seven nsSNPs were found to be highly polymorphic with a risk score of 3-4. The total energies and root-mean-square deviation (RMSD) values were higher for three mutant-type structures compared to the native modeled structure. *Conclusion*. We concluded that three nsSNPs, namely, rs11542041, rs11542040, and rs11542034, to be potentially functional polymorphic.

## 1. Introduction

Alzheimer's disease, the most common form of dementia in the elderly currently, affects more than five million people in the USA alone. Four leading genes (APP, PS1, PS2, and APOE) have been determined, as causative elements of this disorder. It has been seen that the mutations in APP, PS1, and PS2 cause early onset AD while APOE is the only gene that has been always marked as a risk factor for late-onset disease [1–3].

APOE is broadly considered as a crucial agent for AD and is present in 30–50% of patients who develop late-onset AD [4]. It is a circulating 34-kDa secretory protein, synthesized primarily in the liver, and functions in the periphery as a mediator of lipoprotein metabolism through the binding of APOE-containing plasma lipoprotein particles to members of the low-density lipoprotein (LDL) superfamily of receptors. Within the central nervous system (CNS), APOE is synthesized and secreted primarily by astrocytes and microglia, and its importance is underscored by the absence of most other plasma apolipoproteins in brain [5]. Additionally, brain apoE is believed to play a role in the redistribution of lipid and cholesterol during membrane repair and synaptic plasticity as well as in the transport of APOE-containing lipoproteins in the cerebrospinal fluid [6, 7]. The human apoE gene contains several SNPs distributed across the gene [8].

SNPs are the most common polymorphisms of DNA sequence variation for mapping complex genetic traits. About 500,000 SNPs fall in the coding regions of the human genome. Among these, the nonsynonymous SNPs cause changes in the amino acid residues. These are likely to be an important factor contributing to the functional diversity of the encoded proteins in the human population [9]. A number of databases of SNPs are available, such as the human genome variation database, HGVBase [10], and the National Center for Biotechnology Information database, dbSNP [11]. The effect of noncoding SNPs on gene regulation is difficult to understand. So attention is being focused towards nonsynonymous coding SNPs. These types of mutations are believed to be more likely to cause a change in structure and as such alter the function of a protein. These nsSNPs affect gene expression by modifying DNA and transcription factor binding [12, 13] and inactivate active sites of enzymes or change splice sites, thereby produce defective gene products [14, 15].

Epidemiologic association studies focus a great amount of effort into identifying SNPs in genes that may have an association with disease risk. Often, the SNPs that have an association with disease are those that are known as nonsynonymous SNPs, which result in an amino acid substitution. Many molecular epidemiologic studies focus on studying SNPs found in coding regions in hopes of finding significant association between SNPs and disease susceptibility but often find little or no association [16]. With the availability of high-throughput SNP detection techniques, the population of nsSNPs is increasing rapidly, providing a platform for studying the relationship between genotypes and phenotypes of human diseases. Our ability to better select a nsSNP for an association study can be enhanced by first examining the potential impact an amino acid variant may have on the function of the encoded protein with the use of different SNP detection programs like I-Mutant, Sort Intolerant from Tolerant (SIFT), and Polymorphism Phenotype (PolyPhen) [16]. Discovering the deleterious nsSNPs out of a pool of all the SNPs will be very useful for epidemiological population-based studies. So the main aim of this study is to identify deleterious nsSNPs associated with APOE gene.

## 2. Methods

Methodology used was the same as described earlier [9, 16, 17].

### 2.1. Extraction of SNPs

We used dbSNP (http://www.ncbi.nlm.nih.gov/SNP/) to identify SNPs and their related protein sequence for the APOE gene [11]. 

### 2.2. Protein Stability Analysis

I-Mutant 2.0 software was used to predict nsSNP causing protein stability change [18]. I-Mutant 2.0 is a support vector machine- (SVM-) based tool for the automatic prediction of protein stability change upon single amino acid substitution. The protein stability change was predicted from the APOE protein sequence (NP_000032). The software computed the predicted free energy change value or sign (DDG) which is calculated from the unfolding Gibbs free energy value of the mutated protein minus unfolding Gibbs free energy value of the native protein (kcal/mol). A positive DDG value indicates that the mutated protein possesses high stability and vice versa. 

### 2.3. Analysis of Functional Effect on Protein

There are many web-based resources available that allow one to predict whether nonsynonymous coding SNPs may have functional effects on proteins. We chose SIFT [19] to perform protein conservation analysis and predict the phenotypic effect of amino acid substitutions. SIFT is based on the premise that protein evolution is correlated with protein function. Variants that occur at conserved alignment positions are expected to be tolerated less than those that occur at diverse positions. The algorithm uses a modified version of PSIBLAST [20] and Dirichlet mixture regularization [21] to construct a multiple sequence alignment of proteins that can be globally aligned to the query sequence and belong to the same clade. The underlying principle of this program is that it generates alignments with a large number of homologous sequences and assigns scores to each residue, ranging from zero to one. SIFT scores [22] were classified as intolerant (0.00–0.05), potentially intolerant (0.051–0.10), borderline (0.101–0.20), or tolerant (0.201–1.00). the higher the tolerance index of a particular amino acid substitution, the lesser is its likely impact.

### 2.4. Evaluation of Functional Change in nsSNPs

PolyPhen [23] is a computational tool for identification of potentially functional nsSNPs. Predictions are based on a combination of phylogenetic, structural, and sequence annotation information characterizing a substitution and its position in the protein. For a given amino acid variation, PolyPhen performs several steps: (a) extraction of sequence-based features of the substitution site from the UniProt database, (b) calculation of profile scores for two amino acid variants, and (c) calculation of structural parameters and contacts of a substituted residue. PolyPhen scores were classified as “benign” or “probably damaging” [22]. Input options for the PolyPhen server are protein sequence or accession number together with sequence position with two amino acid variants. We submitted the query in the form of protein sequence with mutational position and two amino acid variants. PolyPhen searches for three-dimensional protein structures, multiple alignments of homologous sequences, and amino acid contact information in several protein structure databases. Then, it calculates position-specific independent counts (PSICS) scores for each of two variants and computes the differenceis of the PSIC scores of the two variants. The higher a PSIC score difference, the higher functional impact a particular amino acid substitution is likely to have. PolyPhen scores of >2.0 are expected to be “probably damaging” to protein structure and function, and PolyPhen scores of 1.99–1.50 are expected to be “possibly damaging” to protein function [17].

### 2.5. Analysis of Functional nsSNPs by FASTSNP

The Functional Analysis and Selection Tool for Single Nucleotide Polymorphism (FASTSNP) is a web server which connects many software and databases for processing analysis [24]. We used FASTSNP for the prediction of the functional effect of nsSNPs and an estimation of their risk score. The FASTSNP uses a decision tree for prioritizing the functional effect and estimating risk score. The nsSNPs were submitted for FASTSNP analysis, and output files were displayed as a decision tree.

### 2.6. Modeling of APOE: Its Mutant Forms and RMSD Calculations

Structural analyses are performed based on the crystal structure of the protein for evaluating the structural stability of native and mutant proteins. We used dbSNP to identify the protein coded by APOE gene. The 3D structure of APOE protein was not available, so we used homology modeling approach for its 3D structure prediction. The modeling was performed by automated homology modeling program, SWISS MODEL [25]. The following steps were followed: template structure search using BLAST (http://www.ncbi.nlm.nih.gov/). The FASTA sequence of APOE was submitted to NCBI BLAST. Following BLAST query, a receptor binding domain of human APOE (PDB ID: 2KC3) was selected as template sequence [26]. The template was submitted to SWISS MODEL automated homology modeling server. The validation for structure model obtained from the SWISS MODEL was performed by using PROCHECK [27] and energy minimization performed by Verify 3D [28] and NOMAD-Ref server [29]. The overall stereochemical quality of the protein was assessed by Ramachandran plot analysis [30]. The structures were visualized using Swiss PDB viewer.

The mutation was performed by using Swiss PDB viewer, and energy minimization for 3D structures was performed by NOMAD-Ref server [29]. This server use Gromacs as default force field for energy minimization based on the methods of steepest descent, conjugate gradient and L-BFGS methods [31]. We used conjugate gradient, method for optimizing the 3D structures. Divergence in mutant structure with native structure is due to mutation, deletions, and insertions [32], and the deviation between the two structures is evaluated by their RMSD values which could affect stability and functional activity [33].

## 3. Results

### 3.1. SNP Analysis

The APOE gene investigated in this work contained a total of 88 SNPs, of which 26 were nsSNPs, 8 were synonymous SNPs, and 2 were in noncoding regions, which comprise 1 SNP in the 5′ UTR and 1 SNP in the 3′ UTR. The rest were in the intron region. We selected nonsynonymous coding SNPs for our investigation (Table 1). 

### 3.2. Identification of Functional nsSNP

The more negative the DDG value is, the less stable the given point mutation is likely to be, as predicted by I-Mutant 2.0 server. We obtained 17 nsSNPs that were found to be less stable by this server as shown in Table 1. Out of 17 nsSNPs, 8 nsSNPs, namely, rs121918398, rs121918394, rs41382345, rs11542041, rs11542040, rs11542034, rs11083750, and rs7412 showed, a DDG value of >−1.0. The remaining nsSNPs showed a DDG value of <−1.0, as depicted in Table 1. Out of these 8 nsSNPs, the four nsSNPs, namely, rs121918398, rs11542041, rs11542040, and rs11083750, changed their amino acid from nonpolar to polar two nsSNPs, namely, rs41382345 and rs11542034, changed their amino acids from polar to nonpolar. The other two do not show any change in property. Since the amino acid mutations in these six nsSNPs changed their physiochemical properties, we considered these nsSNPs to be least stable and deleterious by this analysis. 

### 3.3. Prediction of Deleterious nsSNPs

Twenty-six nsSNPs retrieved from APOE gene, submitted independently to the SIFT showed 10 nsSNPs to be deleterious, having the tolerance index score of ≤0.05. The results are shown in Table 1. We observed that, out of 10 deleterious nsSNPs, 5 nsSNPs showed a highly deleterious tolerance index score of 0.00. Among these deleterious 10 nsSNPs, two nsSNPs showed a nucleotide change from C→A, two from G→A, one from A→T, four from C→T, and the other one from A→G (Table 1). Also, according to the SIFT results, the two nsSNPs, namely, rs11542041 and rs11542040, changed their amino acid from nonpolar to polar and two nsSNPs, namely, rs41382345 and rs11542034, changed their amino acid from polar to nonpolar in the mutant protein. We found that these four nsSNPs that are seen to be deleterious according to SIFT were also found less stable by I-Mutant 2.0 server. Therefore, these four nsSNPs were found deleterious by this investigation.

### 3.4. Identification of Damaged APOE nsSNPs

To identify the APOE nsSNPs that affected protein structure, the APOE nsSNPs were analyzed for predicting a possible impact of amino acids on the structure and function of the protein using PolyPhen server. The APOE protein sequence (NP_000032) with each nsSNP position and their 2 amino acid variants was submitted as input for analyzing the protein structural change due to amino acids. Our result showed 9 nsSNPs, namely, rs121918395, rs121918395, rs41382345, rs11542041, rs11542040, rs11542034, rs11542029, rs11083750, and rs7412, to be probably damaging with PSIC score of ≥2.0. The rs41382345, rs11542041, rs11542040, and rs11542034 which were observed to be the cause of protein less stability by I-Mutant 2.0 server and SIFT were also predicted to be probably damaging by PolyPhen server. In addition the other five nsSNPs are highly confidently predicted as probably damaging and the remaining as benign by PolyPhen (Table 1).

### 3.5. Investigation of Functional Effect and Estimated Risk of APOE nsSNPs

In order to identify nsSNP with a high possibility of having a functional effect, FASTSNP was applied for the detection of nsSNP influence on cellular and molecular biological function, for example, transcriptional and splicing regulation. In addition the estimation of risk score was also calculated by FASTSNP. The functional effect and estimated risk of APOE nsSNPs are shown in Table 2. Seven APOE nsSNPs exhibited medium-high-risk score (risk score = 3-4). The functional nsSNPs were rs28931578, rs11542041, rs11542040, rs11542035, rs11542034, rs11083750, and rs7412. The eight nsSNPs showed low-medium risk (risk score = 2-3). The risk score of the remaining ten nsSNPs was unknown. The most important findings detected by FASTSNP were the five nsSNPs, namely, rs28931578, rs11542041, rs11542040, rs11542034, and rs7412 to have high possible functional effect. These were also found polymorphic by I-Mutant 2.0 SIFT as well as by PolyPhen server.

### 3.6. Modeling of Mutant Proteins

**Figure 1:**
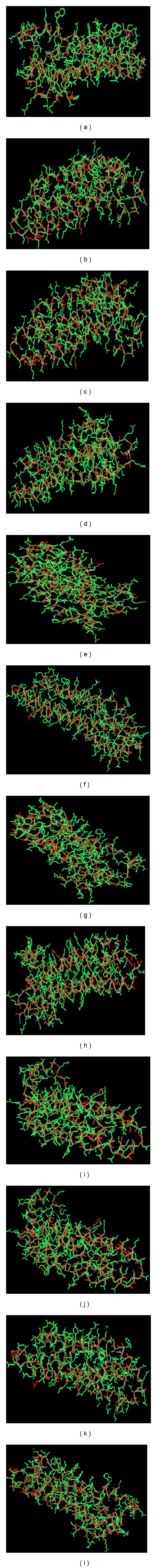
Superimposed homology modeled structure (green) with (a) mutant (E139V), (b) mutant (E150G), (c) mutant (E189K), (d) mutant (K164Q), (e) mutant (P102Q), (f) mutant (P102R), (g) mutant (P102T), (h) mutant (R50C), (i) mutant (R132S), (j) mutant (R152Q), (k) mutant (R154S), (l) mutant (R176C) all in red colour.

**Figure 2:**
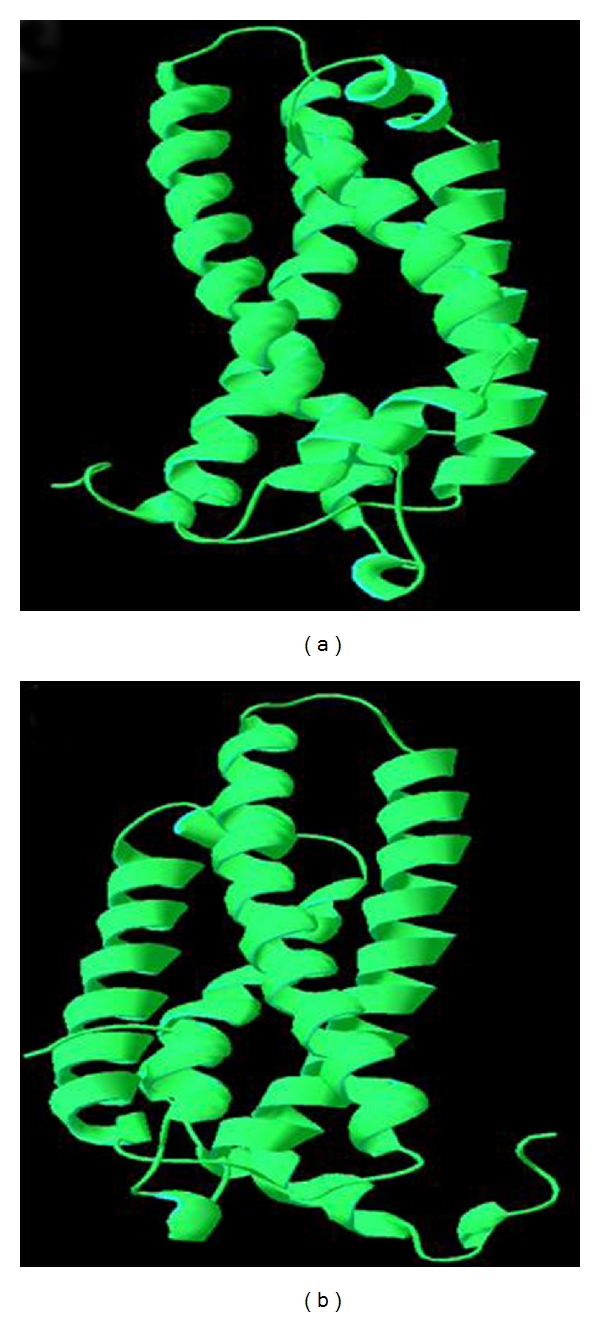
(a) 3D structure of template (PDB ID 2KC3), (b) homology modeled structure of APOE protein predicted using SWISS-MODLE.

The mutations in the APOE were performed by Swiss PDB viewer independently to achieve modeled structures. Then, energy minimizations were performed by NOMAD-Ref server for the homology modeled structure and its mutant forms. The total energy and the RMSD values for the homology modeled structure and the mutant-structures are given in Table 3. The higher the RMSD value is, the more the deviation between the two structures, which in turn changes their functional activity. The total energies and RMSD values were higher for three mutant-structures compared to the homology modeled structure (Table 3); these three nsSNPs could be believed to affect the structure of the proteins. These three nsSNPs were also shown to be deleterious and damaging by I-Mutant 2.0 and SIFT, PolyPhen as well as by FASTSNP server. The structure of template and the newly predicted structure of APOE are shown in Figure 1 while the superimposed structure of homology-modeled protein with its mutant forms is given in Figure 2. 

## 4. Discussion

Our analysis revealed 26 SNPs as nonsynonymous out of which 6 nsSNPs, namely, rs121918398, rs11542041, rs11542040, rs11083750, rs41382345, and rs11542034, were found to be least stable by I-Mutant 2.0 with DDG value of >−1.0. nsSNPs, namely, rs11542041, rs11542040, rs41382345, and rs11542034, showed a highly deleterious tolerance index score of 0.00 with a change in their physicochemical properties by SIFT server. Nine nsSNPs namely, rs121918395, rs121918395, rs41382345, rs11542041, rs11542040, rs11542034, rs11542029, rs11083750, and rs7412, were found to be probably damaging with PSIC score of ≥2.0 by PolyPhen server. Seven nsSNPs, namely, rs28931578, rs11542041, rs11542040, rs11542035, rs11542034, rs11083750, and rs7412, were found to be highly polymorphic with a risk score of 3-4 with a possible effect of nonconservative change and splicing regulation by FASTSNP. The total energies and RMSD values were higher for three mutant-type structures compared to the native modeled type structure. Three nsSNPs are rs11542041, rs11542040, and rs11542034 which were also shown to be deleterious and damaging by I-Mutant 2.0, SIFT, and PolyPhen as well as by FASTSNP server.

A major interest in human genetics is to distinguish mutations that are functionally neutral from those that contribute to disease. Amino acid substitutions currently account for approximately half of the known gene lesions responsible for human inherited disease. Therefore, the identification of nsSNPs that affect protein functions and relate to disease is an important task. The effect of many nsSNPs will probably be neutral as natural selection will have removed mutations on essential positions. Assessment of nonneutral SNPs is mainly based on phylogenetic information (i.e., correlation with residue conservation) extended to a certain degree with structural approaches. However, there is increasing evidence that many human disease genes are the result of exonic or noncoding mutations affecting regulatory regions [17, 34]. Much attention has been focused on modeling by different methods the possible phenotypic effect of SNPs that cause amino acid changes and only recently has interest focused on functional SNPs affecting regulatory regions or the splicing process. Moreover, because of their widespread distribution on the species genome, SNPs become particularly important and valuable as genetic makers in the research for the diseases and corresponding drug [35]. Currently, millions of human SNPs have reported by high-throughput methods. The vast number of SNPs causes a challenge for biologists and bioinformaticians although they provide lot information about the relationships between individuals [35]. Beside numerous ongoing efforts to identify millions of these SNPs, there is now also a focus on studying associations between disease risk and these genetic variations using a molecular epidemiological approach. This plethora of SNPs points out a major difficulty faced by scientists in planning costly population-based genotyping, which is to choose target SNPs that are most likely to affect phenotypic functions and ultimately contribute to disease development [17, 35, 36].

Presently, most molecular studies are concentrating on SNPs located in coding and regulatory regions; yet several of these studies have been unable to identify substantial associations between SNPs and disease vulnerability. To develop a rational approach for prioritizing SNP selection for genotyping in molecular studies, an evolutionary perspective to SNP selection is applied. The assumption is that amino acids conserved across species are more expected to be functionally significant. Therefore, SNPs that alter these amino acids might more probably be related with disease vulnerability. It has been documented that use of the molecular evolutionary approach may be a potent tool for prioritizing SNPs to be genotyped in upcoming molecular epidemiological studies [17]. Therefore, our analysis will provide useful information in selecting SNPs of APOE gene that are likely to have potential functional impact.

## 5. Conclusion

In our analysis, we found out that nsSNPs (rs11542041, rs11542040, and rs11542034) showed less stable, deleterious, probably damaging, and high-risk score by I-Mutant 2.0, SIFT, PolyPhen, and FASTSNP, respectively. The mutant protein structures of these three nsSNPs also showed very high energy and RMSD values compared to the homology-modeled structure. We therefore concluded these three nsSNPs as the potential functional polymorphic. To those conducting large-scale population-based epidemiologic studies, the idea of prioritizing nsSNPs in the investigation of association of SNPs with disease risk is of great interest. The use of these servers to select potentially polymorphic nsSNPs for epidemiology studies can be an efficient way to explore the role of genetic variation in disease risk and to curtail cost. Furthermore, predicted impact of these nsSNPs can be tested with the use of animal models or cell lines to determine if functionality of the protein has indeed been altered.

## Figures and Tables

**Table 1:** I-Mutant, SIFT and PolyPhen results of APOE.

				SIFT		PolyPhen	
SNP ADs	Alleles	Amino acid change	DDG	Tolerance index	Predicted impact	PSIC score	Predicted impact
rs121918399	C/T	R43C	0.66	0.07	Potentially intolerant	1.534	Possibly damaging
rs121918398	A/G	R292H	−2.06	0.06	Potentially intolerant	1.979	Possibly damaging
rs121918397	G/A	R163H	0.05	0.16	Borderline	1.961	Probably damaging
rs121918395	C/T	R246C	−0.48	0.06	Potentially intolerant	2.386	Possibly damaging
rs121918394	A/C	K164Q	−1.28	0.08	Potentially intolerant	1.550	Possibly damaging
rs121918393	C/A	R154S	−0.51	0.10	Potentially intolerant	1.962	Possibly damaging
rs121918392	G/A	E21K	1.17	0.18	Borderline	1.373	Borderline
rs41382345	A/T	E139V	−1.32	0.00	Intolerant	2.246	Probably damaging
rs28931579	A/C	S314R	−0.09	0.12	Borderline	1.343	Borderline
rs28931578	G/A	R152Q	−0.43	0.01	Intolerant	1.772	Possibly damaging
rs28931577	G/A	A117T	0.65	0.06	Intolerant	1.451	Borderline
rs28931576	A/G	T60A	0.57	0.15	Borderline	0.669	Benign
**rs11542041**	**C/A**	**R132S**	−**2.75**	**0.00**	**Intolerant**	**2.204**	**Probably damaging**
**rs11542040**	**C/A**	**P102T**	−**1.20**	**0.00**	**Intolerant**	**2.396**	**Probably damaging**
rs11542035	G/A	R137H	0.83	0.13	Borderline	0.080	Benign
**rs11542034**	**A/G**	**E150G**	−**1.24**	**0.00**	**Intolerant**	**2.284**	**Probably damaging**
rs11542032	G/A	E189K	−0.89	0.01	Intolerant	1.607	Possibly damaging
rs11542030	A/G	Q205R	0.48	0.11	Borderline	0.794	Benign
**rs11542029**	**C/T**	**R50C**	−**0.82**	**0.00**	**Intolerant**	**2.654**	**Probably damaging**
rs11542027	C/T	S215F	0.84	0.01	Intolerant	1.437	Borderline
rs11083750	C/A	P102Q	−1.15	NA	NA	2.396	probably damaging
	C/G	P102R	−1.18	NA	NA	2.621	probably damaging
rs769455	C/T	R163C	0.08	0.00	Intolerant	1.502	Possibly damaging
rs769452	T/C	L46P	−0.11	0.12	Borderline	1.334	Borderline
rs429358	T/C	C130R	−0.07	1.00	Tolerant	0.231	Benign
**rs7412**	**C/T**	**R176C**	−**1.19**	**0.02**	**Intolerant**	**2.654**	**Probably damaging**

Note: nsSNPs which were found to be deleterious by I-Mutant and SIFT as well as PolyPhen are highlighted as bold.

**Table 2:** Functional effect and estimated risk (FASTSNP).

SNP IDs	Alleles	Amino acid change	Possible effect	Risk score
rs41382345	A/T	E139V	Conservative change, splicing regulation	2-3
rs28931579	A/C	S314R	Conservative change, splicing regulation	2-3
**rs28931578**	**G/A**	**R152Q**	**Nonconservative change, splicing regulation**	**3-4**
rs28931577	G/A	A117T	Conservative change, splicing regulation	2-3
rs28931576	A/G	T60A	Conservative change, splicing regulation	2-3
**rs11542041**	**C/A**	**R132S**	**Nonconservative change, splicing regulation**	**3-4**
**rs11542040**	**C/A**	**P102T**	**Nonconservative change, splicing regulation**	**3-4**
**rs11542035**	**G/A**	**R137H**	**Non conservative change**	**3-4**
**rs11542034**	**A/G**	**E150G**	**Nonconservative change, splicing regulation**	**3-4**
rs11542032	G/A	E189K	Conservative change, splicing regulation	2-3
rs11542030	A/G	Q205R	Conservative change, splicing regulation	2-3
rs11542027	C/T	S215F	Conservative change	2-3
**rs11083750**	**C/A**	**P102Q**	**Nonconservative change, splicing regulation**	**3-4**
rs429358	T/C	L46P	Conservative change	2-3
**rs7412**	**T/C**	**C130R**	**Nonconservative change, splicing regulation**	**3-4**
rs11542029	C/T	R50C	NP	
rs769455	C/G	P102R	NP	
rs769452	C/T	R163C	NP	
rs121918399	C/T	R43C	NP	
rs121918398	A/G	R292H	NP	
rs121918397	G/A	R163H	NP	
rs121918395	C/T	R246C	NP	
rs121918394	A/C	K164Q	NP	
rs121918393	C/A	R154S	NP	
rs121918392	G/A	E21K	NP	

NP: No Prediction; nsSNPs which show high risk score are highlighted as bold.

**Table 3:** RMSD and total energy of modeled structure and its mutant forms.

	Total energy (Kcal/mol)	RMSD (Å)
Homology modeled structure	−10003.979	—
Mutant model (E139V)	−9997.772	0.06
**Mutant** **model (E150G)**	−**9576.393**	**2.83**
Mutant model (E189K)	−9788.449	0.42
Mutant model (K164Q)	−10173.771	0.00
Mutant model (P102Q)	−10191.791	0.00
Mutant model (P102R)	−10272.199	0.00
**Mutant** **model (P102T)**	−**9601.084**	**2.35**
Mutant model (R50C)	−9923.915	0.12
**Mutant** **model (R132S)**	−**9537.828**	**2.45**
Mutant model (R152Q)	−9995.500	0.11
Mutant model (R154S)	−9961.830	0.09
Mutant model (R176C)	−9743.661	0.41

Note: nsSNPs which show highest energy and RMSD values are highlighted as bold.
